# Recovery of Bioactive Compounds from Industrial Exhausted Olive Pomace through Ultrasound-Assisted Extraction

**DOI:** 10.3390/biology10060514

**Published:** 2021-06-10

**Authors:** Irene Gómez-Cruz, María del Mar Contreras, Florbela Carvalheiro, Luís C. Duarte, Luisa B. Roseiro, Inmaculada Romero, Eulogio Castro

**Affiliations:** 1 Centre for Advanced Studies in Earth Sciences, Energy and Environment (CEACTEMA), Universidad de Jaén, Campus Las Lagunillas, 23071 Jaén, Spain; igcruz@ujaen.es (I.G.-C.); iromero@ujaen.es (I.R.); ecastro@ujaen.es (E.C.); 2 Department of Chemical, Environmental and Materials Engineering, Universidad de Jaén, Campus Las Lagunillas, 23071 Jaén, Spain; 3 Unidade de Bioenergia e Biorrefinarias, LNEG—Laboratório Nacional de Energia e Geologia, 1649-038 Lisboa, Portugal; florbela.carvalheiro@lneg.pt (F.C.); luis.duarte@lneg.pt (L.C.D.); luisa.roseiro@lneg.pt (L.B.R.)

**Keywords:** exhausted olive pomace, experimental design, ultrasound-assisted extraction, bioactive compounds, hydroxytyrosol, mannitol, valorization

## Abstract

**Simple Summary:**

Exhausted olive pomace (EOP) is the main residue of the pomace oil extraction industry, which is generated in large quantities and has limited applications. Thus, this study aimed to obtain bioactive compounds from EOP using ultrasound-assisted extraction as a potential first valorization step. Two types of devices were tested: bath- and probe-type UAE. The operational parameters were studied and optimized to maximize the antioxidant compounds. In particular, hydroxytyrosol was the main phenolic compound identified and its content was 5.16 mg/g EOP (bath-type UAE) and 4.96 mg/g EOP (probe-type UAE). Mannitol was also detected in the extract, 59.53 mg/g EOP (bath-type UAE) and 69.73 mg/g EOP (probe-type UAE). The results highlight the great potential EOP has as a source of bioactive compounds, with applicability in several sectors. Moreover, the probe-type UAE shows potential to be applied for obtaining these bioactive compounds in a continuous and faster manner.

**Abstract:**

Exhausted olive pomace (EOP) is the main agro-industrial waste of the olive pomace extracting industries. It contains phenolic compounds and mannitol, so the extraction of these bioactive compounds should be considered as a first valorization step, especially if EOP is used as biofuel. Therefore, EOP was subjected to bath-type ultrasound-assisted extraction (UAE), and the effects of the acetone concentration (20–80%, *v*/*v*), solid load (2–15%, *w*/*v*), and extraction time (10–60 min) on the extraction of antioxidant compounds were evaluated according to a Box–Behnken experimental design. By means of the response surface methodology, the optimum conditions were obtained: 40% acetone, 8.6% solids, and 43 min. For all the extracts, the total phenolic content (TPC), flavonoid content (TFC), and antioxidant activity (DPPH, ABTS, and FRAP) were determined. With the aim of shortening the extraction time, a two-level factorial experiment design was also carried out using a probe-type UAE, keeping the solid load at 8.6% (*w*/*v*) and the acetone concentration at 40% (*v*/*v*), while the amplitude (30–70%) and the extraction time (2–12 min) were varied to maximize the aforementioned parameters. Finally, a maximum of phenolic compounds was reached (45.41 mg GAE/g EOP) at 12 min and 70% amplitude. It was comparable to that value obtained in the ultrasonic bath (42.05 mg GAE/g EOP), but, remarkably, the extraction time was shortened, which translates into lower costs at industrial scale. Moreover, the bioactive compound hydroxytyrosol was found to be the major phenolic compound in the extract, i.e., 5.16 mg/g EOP (bath-type UAE) and 4.96 mg/g EOP (probe-type UAE). Other minor phenolic compounds could be detected by capillary zone electrophoresis and liquid-chromatography–mass spectrometry. The sugar alcohol mannitol, another bioactive compound, was also found in the extract, and its content was determined. Thus, the use of this technology can support the valorization of this waste to obtain bioactive compounds, including mannitol, hydroxytyrosol, and other derivatives, before being applied for other uses.

## 1. Introduction

Exhausted olive pomace (EOP) is the residual solid of the olive pomace oil industry obtained after subjecting the olive pomace to a drying process and a solid–liquid extraction with hexane [1]. In Spain, around 1.2 million tons of this byproduct are generated every year [2]. This waste contains a moisture level of around 10%, and it is composed of olive skin, exhausted pulp, and different proportions of pieces of stone [3]. EOP has a high-calorific value, and it is nowadays used as renewable low-cost fuel, while the ashes that are generated can be used for the production of ceramic materials [4]. Both activities produce emissions of dangerous particles and gases during their combustion, thus causing an environmental problem [5]. Therefore, other alternatives for EOP valorization have been published, e.g., the obtainment of sugars, xylitol, and ethanol from their polymeric sugar fraction and lignin [2,3,6,7], as well as the production of xylanases [8]. None of these ways are opposed to the extraction of bioactive compounds, such as phenolic compounds, as a first step in a biorefinery concept, instead of being lost without any revenue. Its extraction from agro-industrial wastes can even solve problems of contamination if these are discarded, while it can improve other valorization steps, like fermentation, where phenolic compounds act as inhibitors [6,9]. In previous studies, our research team has evaluated extraction strategies to recover bioactive compounds from EOP, such using water as a solvent at 85 °C and ethanolic solutions [10,11]. Interestingly, hydroxytyrosol was found in the extracts, which has showed clinical relevance in several studies in humans [12,13], as well as it is one of the active compounds in olive oil [14]. Hydroxytyrosol-containing extracts can also be added to foods such as vegetable oils to increase their oxidative stability [15,16].

The extraction of bioactive compounds from plant raw materials is quite complex because it depends on many factors such as their polarity, hydroxyl groups, aromatic rings, type of solvent, particle size, temperature, and extraction time [17]. According to Şahin et al. [18], it is necessary to optimize specific extraction methods for each type of phenolic compound. Recently, new extraction techniques, such as accelerated solvent extraction, ultrasound-assisted extraction (UAE), and microwave-assisted extraction, have been developed. All these techniques can shorten extraction time and lower solvent consumption compared to conventional methods, such as maceration, Soxhlet extraction, and hydrodistillation [19,20]. In particular, UAE is based on the principle of acoustic cavitation on the propagation of sound waves. The collapse of bubbles can produce chemical, physical, and mechanical effects that cause damage to the cell walls of the plant matrix and lead to the release of bioactive compounds [21,22]. This technology can be applied to obtain different phytochemicals, among which phenolic compounds stand out [23]. Two types of ultrasound equipment are commonly used at the lab scale: ultrasonic baths (indirect sonication) and probes (direct sonication) [24]. Today, UAE is considered a green technique for energy-efficient processes, with very important gains in terms of extraction efficiency and economically on an industrial scale [25,26]. In fact, new advances in the ultrasound field have been developed to meet actual needs [26] and some devices are available at the industrial scale, working in a continuous mode. Thus, the optimization of the extraction conditions at the lab scale is a required step before moving to the industrial scale.

**Figure 1:**
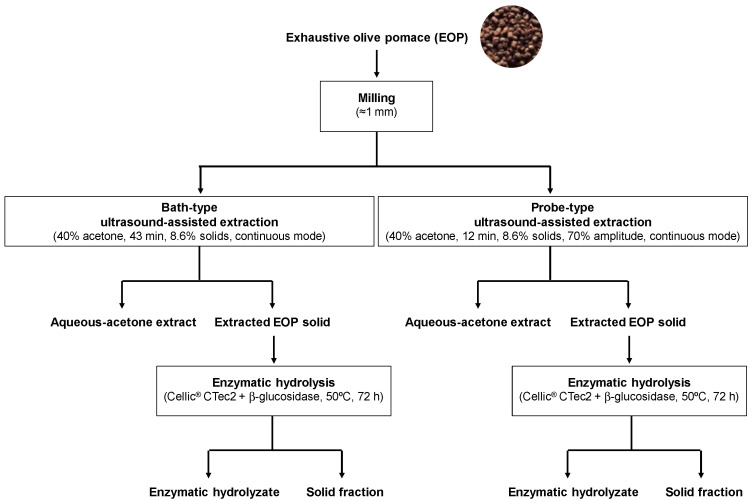
Scheme summarizing the procedures used and samples obtained in this work.

The main objective of this work was to study the influence of three operational variables (extraction time, solid loading, and acetone/water concentration) on the extraction of phenolic compounds from EOP while comparing bath- and probe-type UAE. The response surface methodology (RSM) was applied to optimize the extraction conditions and to recover the maximum total phenolic (TPC) and flavonoid contents (TFC), as well as the antioxidant activity. The extracts obtained at optimal conditions were characterized by capillary zone electrophoresis (CZE), high-performance liquid chromatography (HPLC) coupled to a diode array detector (DAD) and mass spectrometry (MS) for a deeper knowledge of their chemical composition. Finally, two drying methods (freeze-drying and oven-drying) were assessed to obtain dry extracts, and the residual extraction solids were characterized for further valorization. Figure 1 shows the general scheme followed in this work.

Overall, the novelty of this study is the development of a fast extraction method based on UAE to obtain antioxidants, including hydroxytyrosol and mannitol, from a little-explored waste, EOP, as a first step in its valorization within a biorefinery context.

## 2. Materials and Methods

### 2.1. Raw Material and Chemical Characterization

Industrial EOP was obtained from a local olive pomace factory “Spuny SA” (Jaén, Spain), in which EOP is obtained as a partially depitted, pelletized, and dry (moisture content around 6.5%) waste.

In the laboratory, before the extraction process, the sample was milled using a 1 mm screen with an Ultra Centrifugal Mill ZM 200 (Retsch, Haan, Germany). Afterwards, the raw material and the residual extracted EOP solids were subjected to compositional analysis according to the National Renewable Energy Laboratory (NREL) methods [27].

### 2.2. Chemical and Standards

All the chemicals and reagents were of analytical grade and were supplied by Sigma-Aldrich (St. Louis, MO, USA): Folin–Ciocalteu’s phenol reagent, sodium carbonate, sodium nitrite, aluminum chloride, sodium hydroxide, acetic acid, sodium acetate, 2,4,6,-tri(2pyridyl)-1,3,5,-triazine (TPTZ), iron (III) chloride, 1,1-diphenyl-2-picrylhydrazyl (DPPH), sodium chloride, potassium chloride, dipotassium hydrogen phosphate, disodium hydrogen phosphate, ABTS [2,2′-azino-bis(3-ethylbenzothiazoline-6-sulfonic acid) diammonium salt, Trolox (6-hydroxy-2,5,7,8-tetramethylchroman-2-carboxylic acid), and standards of gallic acid and rutin. Methanol (HPLC grade) was obtained from Honeywell (Morristown, NJ, EEUU), pure acetone (pharma grade), and acetonitrile (HPLC grade) from PanReac AppliChem (Barcelona, Spain). Hydroxytyrosol (98% of purity, *w*/*w*) was procured from Extrasynthese (Lyon, France). Ultrapure water was obtained using a Milli-Q system (Millipore, Bedford, MA, USA).

### 2.3. Ultrasound-Assisted Extraction

Firstly, the solid–liquid extraction of EOP was performed in an ultrasonic bath (Ultrasonic, J.P. Selecta, Barcelona, Spain), which was operated in continuous mode at a power of 100 W and a frequency of 40 kHz. The milled EOP was added to different acetone–water solutions in 250 mL ISO flasks (POBEL, Madrid, Spain). The total extraction yield, TPC, TFC, and antioxidant activity were evaluated. The EOP acetone extraction was performed according to a Box–Behnken experimental design (BBD) with 17 experiments in random order, including five central points, which allowed for the determination of the optimal extraction conditions based on the desirability function. As operational variables, the effect of the solid loading (2–15%, *w*/*v*), extraction time (10–60 min), and acetone concentration (20–80%, *v*/*v*) were studied (Table 1). The natural and coded values of these factors are presented in Table 1.

In addition to the control of the conditions at room temperature (without the ultrasonic action and without agitation) was carried out to check the efficiency of the ultrasonic extraction at the conditions of the central points (8.5% solid loading, 35 min, and 50% acetone).

The samples were not cooled; therefore, although the experiments were initiated in the bath at room temperature (26 ± 4 °C), the temperature increased during extraction due to the effects of sonication (up to 46 °C). Accordingly, the temperature reached at the end of each assay was measured. After each extraction, the samples were vacuum-filtered, and around 76% of the volume was recovered. An aliquot of the extracts was filtered with a syringe filter (nylon; 0.45 μm pore size) (SinerLab Group, Madrid, Spain) and stored at −20 °C until analysis. A portion of the extracts was dried at around 45 °C in an oven (Memmert, Schwabach, Germany) for 24 h, and another one was directly frozen and freeze-dried till room temperature using a Noxair freeze-drier (Barcelona, Spain). The remaining powder was redissolved in a 40% acetone solution using the same volume evaporated before analysis.

Additionally, to obtain the extraction yield, another portion of 1 mL of each extract was dried at 105 °C to constant weight. All samples were measured in triplicate, and the extraction yields were expressed as g of extract/100 g of EOP.

Moreover, based on the optimal conditions obtained by the BBD and to shorten the extraction time, a two-level factorial design (FD) was performed on a probe-type ultrasound (Branson SFX150, Ultrasonics Corporation, Brookfield, CT, USA) (power: 150 W; frequency: 40 kHz) working in a continuous mode. For the extraction, 250 mL ISO bottles were used as before, and a 3.17 mm diameter microtip was immersed 1 cm deep into the sample. For this purpose, the solid loading and the acetone percentage were set at the optimal conditions and the amplitude (30–70%) and extraction time (2–12 min) were varied. The temperature at the beginning and the end of the assays was also measured, with a maximum increment of 26 °C (Table S1). The extracts were filtered as before for further analyses, and a mean volume of around 75% was recovered.

### 2.4. Characterization of EOP Extracts: Total Phenolic and Flavonoid Content and Antioxidant Activity

#### 2.4.1. Total Phenolic and Flavonoid Content

The TPC was determined using the Folin–Ciocalteu colorimetric assay, according to a procedure described by Singleton and Rossi [28] with some modifications: 3 mL of Folin–Ciocalteu reagent were added to 0.3 mL of diluted extract, followed by 2 mL of a solution of Na_2_CO_3_ (10% *w*/*v*). After agitation and 1 h in the dark at room temperature, the absorbance was measured at 760 nm. Gallic acid was used as standard, and the results were expressed as g/L in the extract and mg of gallic acid equivalents (GAE)/g of EOP.

The TFC was measured according to a colorimetric method reported by Blasa et al. [29]: 1 mL of diluted extract was added to 0.3 mL of a solution of NaNO_2_ (5%, *w*/*v*), followed by 0.3 mL of a 10% AlCl_3_ solution after 5 min, and the resultant solution was mixed. Six minutes later, 2 mL of a 1 M NaOH solution were added, and the resultant solution was mixed. After 5 min (in the dark at room temperature), the absorbance was measured at 510 nm. Rutin was used as the reference standard, and the results are expressed as mg of rutin equivalents (RE)/g EOP.

All the measurements were carried out in triplicate, and a Bio-Rad iMarkTM microplate absorbance reader was employed (Hercules, CA, USA) with 96-well transparent polystyrene microplates.

#### 2.4.2. Trolox Equivalent Antioxidant Capacity Assays

Three different assays were used to determine the antioxidant activity of the extracts: DPPH and ABTS™ radical scavenging assays and ferric-reducing power assays (FRAP), as described by Martínez-Patiño et al. [30]. In brief, in the DPPH radical scavenging assay, 2 mL of methanolic solution of DPPH were added to a 200 µL of sample. The sample was shaken and kept in the dark for 15 min, and then its absorbance was measured at 517 nm. In the ABTS radical scavenging assay, an ABTS stock solution (7 mM) with 2.45 mM potassium persulfate was diluted with a phosphate buffer (pH = 7.4) to an absorbance of 0.7 at 734 nm. Then, 3 mL of this solution were added to 30 µL of sample, and after 6 min, the absorbance was measured at 734 nm. In FRAP, a 10:1:1 solution was prepared with a 300 mM acetate buffer (pH = 3.6), a 10 mM TPTZ solution in 40 mM HCl, and 20 mM FeCl_3_∙6H_2_O in distilled water. This reagent (3 mL) was added to 100 µL of sample, and the absorbance was measured at 593 nm after 6 min.

For all three assays, Trolox was used as standard for comparison, and the results were expressed as mg of Trolox equivalents (TE)/g of EOP. All the measurements were carried out in triplicate and the aforementioned microplate absorbance reader, and plates were employed.

### 2.5. CZE, RP-HPLC-DAD and RP-HPLC- Mass Spectrometry (MS) Analyses

#### 2.5.1. Phenolic Compounds

The phenolic profile of the extracts was obtained by CZE using a Capillary Electrophoresis (CE) system from Agilent Technologies (Waldbronn, Germany), equipped with a DAD, using an Agilent uncoated fused silica capillary (50 μm) with an effective length of 62/56 cm. The separation buffer was 15 mM sodium tetraborate decahydrate with 8% methanol and adjusted to pH 9.1. The separation voltage was 30 kV with a ramp of 0.5 min, the current was at 120 μA maximum setting, and the capillary temperature was set at 30 °C. The samples were injected using a pressure of 50 mbar for 5 s into the anode (+) of the CE system. Between runs, the capillary was pre-conditioned by washing with 0.1 M NaOH (3 min) followed by the buffer (3 min). Agilent 3D-CE ChemStation data software (Rev B.04.01) was used to perform qualitative analysis by comparison of the migration time and UV spectra of samples with the ones of authentic standards run in the same conditions and stored in an in-house library.

Reversed phase (RP)-HPLC-DAD analyses were performed in a Shimadzu Prominence device equipped with a DGU-20A5 degasser, LC-20AD quaternary pump, SIL-20AC HT auto sampler, SPD-M20A DAD, and CTO-10AS VP column oven (Kyoto, Japan). The analysis was performed according to the work of Lama-Muñoz et al. [19] using a BDS HYPERSIL C18 column (4.6 mm × 250 mm, 5 μm particle size) (Thermo Fisher Scientific Inc., Waltham, MA, USA). The mobile phases were a Milli-Q^®^ water/0.2% orthophosphoric acid (solvent A), methanol (solvent B), and acetonitrile (solvent C). The initial composition was 96/2/2 (*v*/*v*/*v*), and then the gradient elution was as follows: B and C changed from 2 to 25% in 40 min, 25 to 30% in 5 min, 30 to 50% in 15 min, isocratic at 50% for 8 min, and then 50 to 2% in 4 min. The column was equilibrated for 8 min at starting conditions before each injection. The flow rate was 1.0 mL/min, and the injection volume was 20 μL. A hydroxytyrosol calibration curve was obtained at 280 nm (1.25–500 mg/L; R^2^ > 0.999).

RP-HPLC–MS and MS^2^ analyses were performed in an Agilent 1100 HPLC connected on-line to an ion trap (IT) (Esquire 6000; Bruker, Bremen, Germany) via an electrospray interface, following the work of Medfai et al. [31]. The flow rate was 0.35 mL/min, and the injection volume was 10 L. The mobile phases were: Milli-Q^®^ water and formic acid (0.1%, *v*/*v*) as solvent A and acetonitrile and formic acid (0.1%, *v*/*v*) as solvent B; a linear gradient of solvent B in A was used: B changed from 4 to 7% in 1 min, 7 to 30% in 15 min, 30 to 40% in 4.5 min, 40 to 100% in 4.5 min, isocratic at 100% for 2 min, 100 to 4% in 1.5 min, and isocratic at 4% for 7 min. A Kinetex core-shell C18 column (2.1 mm × 50 mm, 2.7 m) (Phenomenex, Barcelona, Spain) was applied. MS and MS/MS spectra were recorded over the mass-to-charge (*m*/*z*) range of 100–1200 in the negative ionization mode, and 4 spectra were averaged. Auto MS/MS analyses were performed at 0.6 V. The data were processed using DataAnalysis (version 4.0) from Bruker.

#### 2.5.2. Mannitol and Glucose

Mannitol and glucose contents were determined according to the work of Gómez-Cruz et al. [7] and using an HPLC 1260 series system connected with a refractive index detector (RID) (Agilent Technologies). An ICSep ICE-COREGEL 87 H3 column (Transgenomic, Inc., Omaha, NE, USA) was applied, the temperature was set at 65 °C, and the mobile phase flow was 0.6 mL/min (5 mM sulfuric acid).

### 2.6. Enzymatic Hydrolysis

The residual extracted EOP solids obtained after UAE treatments were washed with water to remove residual acetone and vacuum-filtered. Then, the solids were subjected to enzymatic hydrolysis according to Gómez-Cruz et al. [32] for 72 h using the commercial enzyme solution Cellic^®^ CTec2 (Novozymes A/S, Bagsværd, Denmark) at 15 FPU/g substrate and glucosidase (Novozymes A/S) at 15 IU/g substrate. The experiments were performed in triplicate, and the released glucose was determined by HPLC-RID analysis (Section 2.5.2).

### 2.7. Statistical Analysis

The experimental data obtained after applying the designs were analyzed using the Design-Expert^®^ v8.0.7.1 software (Stat-Ease, Inc., Minneapolis, MN, USA) and RSM. ANOVA was used to determine the significance of the results. The extraction tests were performed in random order.

For multiple comparison analysis, an ANOVA test with Scheffe’s post hoc procedure was applied to compare the means (*p* < 0.05) using Statgraphics Centurion XVII (StatPoint Technologies, Inc., Warranton, VA, USA). In addition, for two comparison analyses, a *t*-test and an F-test were performed using Microsoft Office Excel 2007 (Redmond, WA, USA). The significance level was set at 0.05.

## 3. Results and Discussion

### 3.1. Optimization of Bath-Type UAE

#### 3.1.1. Effect of UAE and Milling on the Recovery of Phenolic Compounds

Acetone–water was selected as the extraction solvent since previous results showed that richer extracts in phenolic compounds can be obtained with this solvent compared to simply water [32]. Acetone can be used in the manufacturing process to obtain food ingredients [33] because it is generally recognized as safe (GRAS) and complies with good manufacturing practices. Then, some preliminary experiments were performed at 8.5% solid loading, 35 min, and 50% acetone (central points of the BBD) using pelletized and milled EOP, with and without the application of ultrasound (Table 2).

It was evident that milling improved the TPC, TFC, and antioxidant activity in both cases, i.e., with or without using ultrasound, with an increase higher than 100%. Moreover, the use of ultrasound favored the extraction, with an increase higher than 60 and 20% in the case of pelletized and milled EOP, respectively, when compared to their respective controls (Table 2). In view of these results, EOP was milled for the optimization study using UAE.

#### 3.1.2. Fitting the Model

The BBD was used to study the effect of the variables of acetone concentration, solid loading, and extraction time on EOP when using bath-type UAE. The extraction yield, phenolic concentration, TPC, TFC, and the antioxidant activity of the extracts were chosen as responses. The experimental results obtained for each response variable are shown in the Table 3.

Then, multiple regression fitting was applied to obtain quadratic polynomial equations that describe the relationship between each response and the three independent variables. Table 4 summarizes the different statistical parameters obtained for the models and the model adjustment to the experimental data. The quality of the fit of the response surface models was assessed by ANOVA. The developed models presented determination coefficients (R^2^) and adjusted determination coefficients (R^2^adj) in the range of 0.933–0.994 and 0.903–0.997, respectively, suggesting that the experimental data matched well with the predicted values. Additionally, the coefficient of variation (CV) was 2.02–5.31% and no lack of fit was observed, which indicated the accuracy and reliability of the model. The outcomes of ANOVA showed high F-values for all response variables (31.33–807.84) with *p*-values lower than 0.05, implying that the models were highly significant. These results confirmed that the suggested models were suitable for forecasting the relationship between the operational variables and the different responses.

#### 3.1.3. Response Surface Analysis

##### Influence of Extraction Conditions on the Extraction Yield, Phenolic Concentration, TPC and TFC

**Figure 2:**
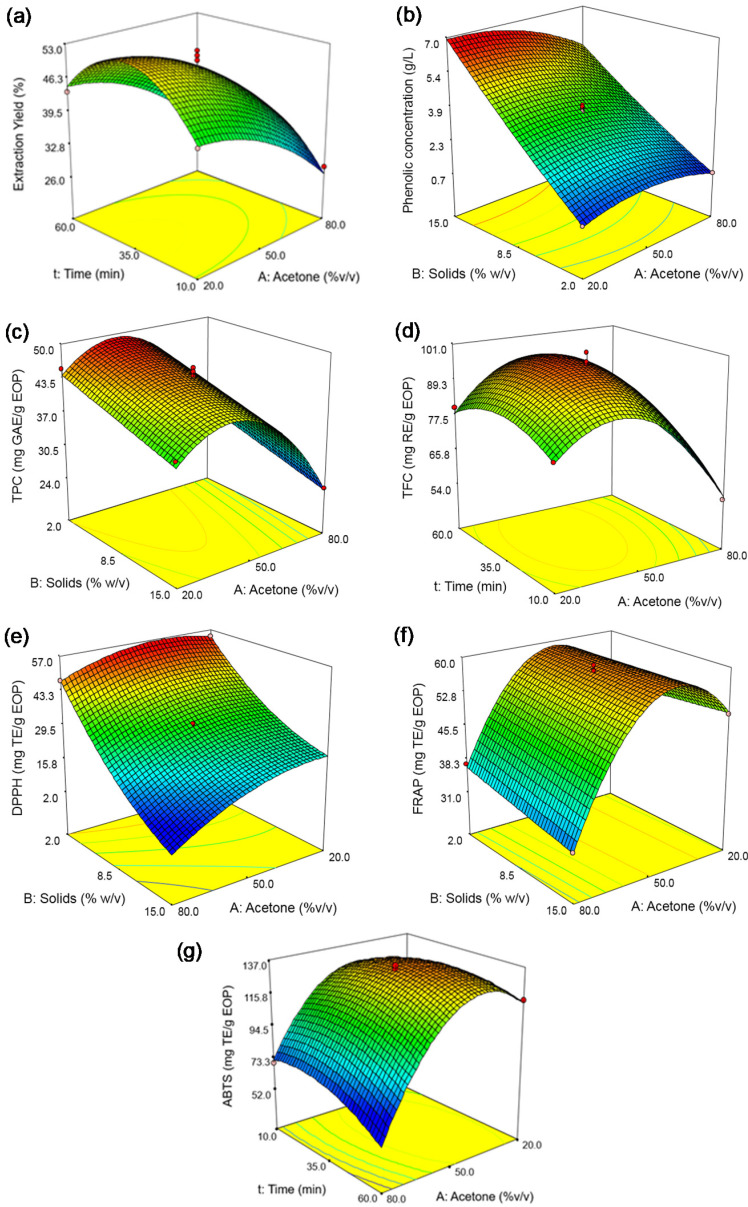
Response surfaces obtained by the Box–Behnken design for exhausted olive pomace: (**a**) extraction yield, (**b**) phenolic concentration, (**c**) total phenolic content (TPC), (**d**) total flavonoid content (TFC), (**e**) DPPH assay, (**f**) FRAP assay, and (**g**) ABTS assay. The solid loading was fixed at 8.5% in plots (**a**,**d**,**g**), and the time at 35 min in plots (**b**,**c**,**e**,**f**).

The experimental values of the extraction yields varied between 26.84 and 58.62% (Table 3). According to the mathematical model of extraction yield (Table 4, Equation (1)), the solid loading, acetone concentration and time were significant factors for this response. As an example, Figure 2a shows that the maximum extraction yield was reached at the intermediate conditions of acetone concentration and time, the former being less pronounced (Figure 2a).

The latter factors play roles in extraction efficiency, but their effects depend on the compound type and the operational conditions [23,34]. For example, a general trend is that the sonication time initially increases the yield, and the yield subsequently decreases when the time is extended, giving a maximum [34,35], as occurred in this work. In the first step, the cavitation effect of the ultrasound may enhance the swelling, hydration and pore formation of the plant tissue, and thereby an increase in the exposure of the solutes occurs, helping their release into the solvent [34]. Secondly, the exposure of ultrasound for very long duration can damage the solutes and decrease the extraction yield. Nonetheless, this behavior is general, and other authors have revealed a linear effect on the total extraction yield for grapes in the range between 11.6 and 28.4 min [36].

Phenolic concentration ranged from 0.74 to 6.52 g GAE/L (Table 3). Equation (2) shows that this response depended almost exclusively on the solid loading having a positive influence (Table 4). As an example, Figure 2b shows the relationship between solid loading and acetone concentration, and a negative interaction is shown. As for other studies, the highest values of phenolic concentration using maceration have been found at intermediate concentrations of acetone, from 40 to 60%, using other biomasses, e.g., 0.48 g GAE/L were obtained from black rice (5% of solid loading) at 40% acetone [37], 0.12 g GAE/L from peach (around 3.3% of solid loading) at 60% acetone [38], and 6.87 mg GAE/g from bunga kantan inflorescence (2.5% of solid loading) at 50% acetone [39]. These authors suggested that extraction efficiencies of these acetone solutions are related to their polarity and viscosity compared to other solvents. Moreover, these results also indicated that EOP is an interesting source of phenolic compounds due to the high amounts that can be recovered compared to those studies.

The TPC varied between 24.09 and 49.03 mg GAE/g EOP (Table 3). As before, the model equation revealed that the acetone concentration was the most influential factor in this response, as can be deduced from the higher coefficients of the linear and quadratic terms for this variable (Equation (3)). These terms were negative, and a maximum can be observed in Figure 2c. Time had a positive influence on this response. Regarding the solid loading, Figure 2c shows that an increase in solid loading produced a decrease in TPC, especially at high acetone concentrations, and a maximum could be achieved at intermediate acetone concentrations and low solid loading values.

The values of TPC are comparable to that of olive tree biomasses [11,30,40], including commercial olive leaves, e.g., the phenolic content ranged between 20.6 and 108 mg/g dry biomass. Olive leaves are currently used to obtain marketable functional extracts (powdered and liquid extracts), which showed a high heterogeneity in the levels of phenolic compounds (7.5–250 mg/g of extract) and oleuropein represented up to 94% of total phenolic compounds [31,41]. In this work and considering the experimental assays in Table 3, the values could vary between 63.1 mg/g of extract (run 12) and 112.1 mg/g of extract (run 5), which was also in the range of the latter values.

Moreover, the values of TFC varied between 54.88 and 100.04 mg RE/g EOP in the performed experiments (Table 3). Similarly to previous responses, the coefficient of the linear term and the quadratic term of acetone concentration were negative and showed the highest influence (Equation (4), Table 4). The solid loading had no important influence on TFC. The highest value for this response was reached by operating at intermediate acetone concentration and time, as Figure 2d shown for the formers factors (at 8.5% solid loading). Overall, the extraction time had different trends depending on the response variable, e.g., only its linear term had a remarkable effect on the TPC, while for the extraction yield and TFC, both the linear and the quadratic terms had influence and a maximum was reached. This could be explained by the fact that flavonoids could be more negatively affected by the ultrasound treatment than other phenolic constituents; as commented before, it depends on the type of compound [34].

##### Influence of Extraction Conditions on the Antioxidant Activity

Three methods were applied to determine the antioxidant activities of the EOP extracts. The experimental values (Table 3) varied between 9.99 and 56.74 mg TE/g EOP in the DPPH assay, between 28.96 and 63.18 mg TE/g EOP in the FRAP assay, and between 69.69 and 144.78 mg TE/g EOP in the ABTS assay. The software generated similar model equations for the DPPH, FRAP, and ABTS assays; i.e., Equations (5–7), respectively. The antioxidant activity depended on the three variables studied, although for the FRAP and ABTS assays, the linear and quadratic terms for acetone concentration were the most significant and had a clear negative influence. The linear term of the solid loading was the most influential factor in the case of the DPPH assay and negatively affected it, as shown Figure 2e. The linear term of the extraction time was more significant for the FRAP assay, showing a positive influence on this response.

Figure 2e,f represents the combined effect of biomass loading and acetone concentration on the antioxidant capacity measured by DPPH and FRAP, respectively, establishing an extraction time of 35 min. As another example, Figure 2g represents the effect of the extraction time and acetone concentration on ABTS assay at 8.5% solid loading. In all cases, a maximum was achieved for the acetone concentration, lowering the antioxidant capacity when a higher concentration was used, as before. In addition, the highest values of antioxidant capacity were reached at the lowest level of biomass loading.

##### Process Optimization and Validation of the Model

An optimization of the three studied variables—acetone concentration, extraction time, and solid loading—was carried to simultaneously maximize the seven measured responses, i.e., yield extraction, phenolic concentration, TPC, TFC, and antioxidant activity determined by the DPPH, ABTS, and FRAP assays. The optimal conditions predicted by the model were: 40% acetone concentration, 8.6% solid loading, and 43 min of extraction time. Table 5 shows the values predicted by the model for all responses under the optimal conditions. The experimental data obtained after reproducing these conditions were similar to the predicted values; the error was less than 10% in all cases. The mean temperature reached was 41 °C, which corresponded to a temperature increment of 15 °C reached in 43 min.

The extraction yield was 47.32% when using 40% acetone. Moreover, the optimized conditions yielded an extract with TPC and TFC values of 3.62 g GAE/L and 7.87 g RE/L, which corresponded to 42.05 mg GAE/g EOP and 91.59 g RE/g EOP, respectively (Table 5). These values were closer to those obtained using water as extraction agent, but 85 °C and 90 min were required in this method, yielding an extract with TPC and TFC values of 44.5 mg GAE/g EOP and 114.9 mg RE/g EOP, respectively [10]. Thus, UAE using 40% acetone can shorten the extraction time without the heating requirement.

### 3.2. Optimization of the Probe-Type UAE

#### 3.2.1. Fitting the Model

After fitting the bath-type UAE of EOP (40% acetone, 43 min, and 8.6% solids), a probe-type UAE was tested with the idea of shortening the extraction time according to previous studies [42]. For this reason, the solid loading (8.6% solids, *w*/*v*) and the acetone percentage (40%, *v*/*v*) were fixed according to the previous optimized conditions and a two-level factorial design with five central points was proposed. In this case, the influence of two operational variables was studied: the amplitude (30–70%) and time of extraction (2–12 min) (Table 1). This design consisted of 12 experiments, which were performed in random order. The same responses that for the BBD were studied (extraction yield, phenolic concentration, TPC, TFC, and antioxidant activity). The experimental results obtained for each response variable are shown in Table 6.

The ANOVA results for each of the responses were statistically significant, with *p*-values < 0.05 in all cases (Table 7) except for the DPPH model. The CV values, which were between 2.38 and 7.50% in the seven response equations, confirmed that the models were precise. The lack of significance was also shown for the models with *p*-values for the lack of fit higher than 0.05 in all cases, indicating that the dispersions of the experimental results were independent of the pure errors of the models. R^2^ and adjusted R^2^ values were also generally adequate.

The experimental results were adjusted to linear regression equations. The equations for the coded values of the independent variables that modeled the seven studied responses are presented in Table 7, with non-significant terms (*p*-values > 0.1).

#### 3.2.2. Response Surface Analysis

For probe-type UAE, the experimental values of the extraction yields varied in a range from 43.39 to 60.74% using the lowest (run 7, 30% amplitude, and 2 min) and highest intense conditions (run 3, 70% amplitude, and 12 min), respectively (Table 6). This also occurred for the rest of the response variables, with the exception of the DPPH assay, in the following range: phenolic concentration (2.94–4.32g GAE/L), TPC (34.24–50.27 mg GAE/g EOP), TFC (81.31–113.63 mg RE/g EOP), FRAP (53.84–76.73 mg TE/g EOP), and ABTS (100.51–144.78 mg TE/g EOP) assays. It can be noted that in the case of the DPPH assay, the values of antioxidant activity varied in a narrow range of 31.53–35.10 mg TE/g EOP (runs 9 and 3, respectively) under the conditions tested.

**Figure 3:**
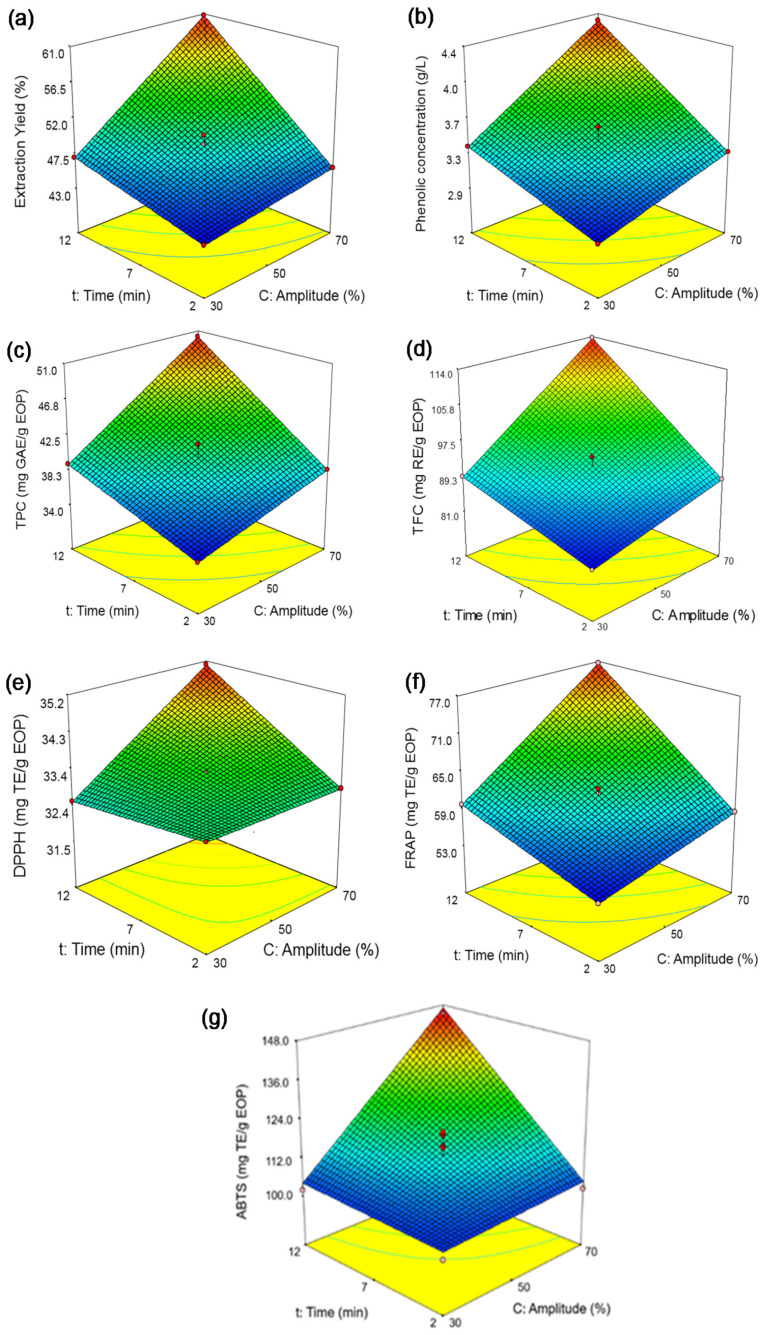
Response surfaces obtained by the Box–Behnken design for exhausted olive pomace: (**a**) extraction yield, (**b**) phenolic concentration, (**c**) total phenolic content (TPC), (**d**) total flavonoid content (TFC), (**e**) DPPH assay, (**f**) FRAP assay, and (**g**) ABTS assay.

According to the mathematical model of extraction yield (Equation (8)), the linear terms of the amplitude and the extraction time had similar weights in this response, and a positive interaction between each one was also observed. This trend was also observed for the rest of variables (Equations (9)–(14)). As an example, Figure 3a–g represent the combined effect of amplitude and extraction time on response variables, with maximums at the highest extraction time and amplitude of 12 min and 70%, respectively. This behavior also indicated a clear correlation between the presence of phenolic compounds and the antioxidant activity of the EOP extracts.

It has been suggested that amplitude and extraction time are key for intensifying the extraction of compounds [23]. In the former case, the resonant bubble size is proportional to the power of the ultrasonic wave and thereby to the amplitude percentage (rated power of the device). As the bubble size increases, their impact on implosion also intensifies, provoking fragmentation, pore formation, and mixing, which enhance the diffusivity and extraction yield. Hydrodynamic force may also be increased, which is related to the disruption of plant tissues [34]. In some cases, high amplitudes may result in solvent agitation instead of cavitation, or, alternatively, an saturation effect may occur due to the cavitation bubbles being assembled around the probe tip [34]; as a consequence, a low transmission of the ultrasounds occurs. Under the conditions tested here, neither of these effects nor the degradation of phenolic compounds, which was another effect observed by other authors when the amplitude is increased, seemed to be observed [43]. The extraction time had a similar effect to the power, as Kumar and coworkers [34] suggested, since a longer extraction time implies a higher exposure of the compounds to the extraction medium and helps their release into the solvent. Nonetheless, this trend was only observed when using the probe-type UAE and not the bath-type UAE. Due to the device restrictions, the extraction time could not be extended in the probe-type UAE and thereby whether longer times could reduce the bioactive compounds could not be evaluated.

##### Validation of the Model

Once the influence of the two factors, amplitude and extraction time, on all responses was analyzed, the best conditions were estimated to simultaneously maximize all of them. The conditions were obtained at 70% amplitude and 12 min for 40% acetone and 8.5% solid loading. These conditions were experimentally reproduced in triplicate to validate the model, and the results are shown in Table 5. It is worth highlighting that the values obtained for the phenolic concentration (3.91 g GAE/L), TPC (45.41 mg GAE/g EOP), TFC (100.81 mg RE/g EOP), and antioxidant activity (35.53–137.16 mg TE/g EOP) were slightly higher to those values obtained using the bath-type UAE, as well as in a shorter time—12 min versus 43 min. This shortening effect agreed with previous studies on grape [42], sunflower seed cake [44], and olive tree biomass [30], with UAE extractions times ranging from 3 to 15 min. The latter authors showed the best conditions to recover oleuropein from olive leaves were 40 °C and 30% of amplitude (in around 15 min) using 60% ethanol and probe-type UAE.

According to Zardo et al. [44], the amplitude (which is the maximum height of a sound wave and so is related to the ultrasound intensity) and time are not the only factors acting, as the temperature could also affect the results. It should also be noticed that ultrasound provokes the formation of small bubbles, as mentioned before, which are subjected to fast adiabatic compression and expansion, thus generating a fast local increase of temperature and pressure [43]. At the selected conditions, the mean reached temperature (45 °C) and the increment of temperature (21 °C reached in 12 min) were higher than those measured using the bath-type UAE (see Section Process Optimization and Validation of the Model). Nonetheless, in our designs, the increment of temperature could be interpreted as a response and the influence of the other operating factors could be studied. While in the former design, the increment of the temperature was mainly positively related to the extraction (or sonication) time (*p*-value < 0.05), in this design, the linear terms of the extraction (or sonication) time, the amplitude, and their interaction (*p*-value < 0.05) were the significant factors that affected this factor in a positive manner (Table S2; Figure S1). Thus, it was difficult to separate the effect of the time and the amplitude from that of the temperature in our work.

### 3.3. Profiles and Standardization

#### 3.3.1. CZE-DAD, HPLC-DAD, and HPLC-RID

**Figure 4:**
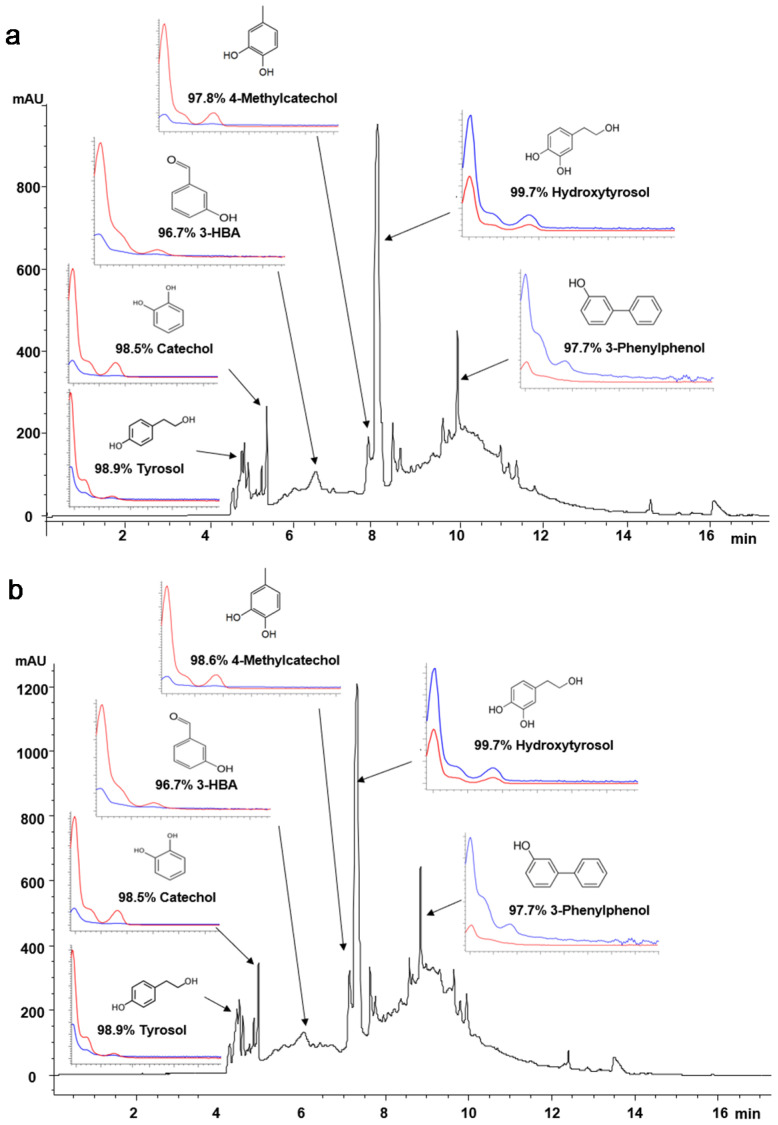
Electropherograms at 200 nm of the water–acetone extract obtained by (**a**) bath- and (**b**) probe-type ultrasound-assisted extraction of exhausted olive pomace in the best obtained conditions.

The extracts obtained at the best conditions using both bath- and probe-type UAE were analyzed by two complementary methods, CZE-DAD (Figure 4) and RP-HPLC-DAD (Figure S2). As can be observed in both figures, the extracts showed similar phenolic profiles.

Figure 4 also shows the complexity of the water–acetone extracts and the phenolic compounds identified at >96% matching with authentic standards: hydroxytyrosol, tyrosol, catechol, 3-hydroxybenzaldehyde, 4-methylcatechol, 3-phenylphenol, and 3-hydroxybenzoic acid or derivatives. Among them, hydroxytyrosol, which is also considered one of the most powerful antioxidants in olive-derived products, was the major compound found in all the extracts. Obtaining this compound from natural low-cost resources is highly interesting due to its clinical relevance [12,13], e.g., hydroxytyrosol and vitamin E have been found to reduce the systemic inflammation and improve steatosis and hypertriglyceridemia in children with non-alcoholic fatty liver disease [12]. When added to vegetable oils, hydroxytyrosol can increase their oxidative stability [15,16], while in olive oil, the hydroxytyrosol cluster contributes to its health benefits [14]. Thus, the content of hydroxytyrosol was estimated using RP-HPLC-DAD at 280 nm, obtaining a slightly higher value for the extract obtained by bath-type UAE compared to the probe-type UAE; i.e., 5.16 ± 0.10 mg HT/g EOP and 4.96 ± 0.03 mg HT/g EOP, respectively. These values were similar to the hydroxytyrosol derivative contents determined in a liquid fraction obtained from olive pomace (5 mg/g powdered liquid) [45] and higher that the content obtained after the treatment of olive pomace at 80 °C for 90 min with 1 M of H_3_PO_4_ (1.36 mg of hydroxytyrosol/g of fresh olive pomace) [46]. Thus, it is again worth noting that EOP is a natural source of hydroxytyrosol, which is highly resistant to the storage and processing conditions that olive pomace is subjected to in the industry to generate pomace olive oil and EOP.

Moreover, the mannitol content of these two samples was also analyzed by HPLC-RID, since our previous results had shown that this compound can pass to the solvent during extraction [7,11]. Mannitol also has biological and food preservative properties (increasing food shelf life by reducing sugar crystallization), as well as being a low-calorie sweetener [1,45], and so its co-extraction had to be confirmed. In fact, its content was 59.53 ± 0.47 mg/g EOP for bath-type UAE and 69.73 ± 2.07 mg/g EOP for probe-type UAE, revealing that EOP is a natural source of this sugar alcohol through olive pomace [45] and olive leaves [47].

#### 3.3.2. HPLC–MS and Tandem MS

The extracts were also analyzed by RP-HPLC–MS and MS/MS since they are powerful tools for the characterization of phenolic compounds, as our previous studies highlighted [11,31]. The characterization work was based on the latter studies [11,31] and a study by Ammar et al. [48], which conducted the exhaustive characterization of olive-derived biomasses using ion trap and/or quadrupole-time of flight. Table 8 and Figure S3 show that, besides hydroxytyrosol, the extracts contained 18 derivatives of hydroxytyrosol and tyrosol. The hydroxytyrosol cluster found in the water–acetone extracts was composed of free forms that are not linked to hydroxycinnamic acids or secoiridoids (hydroxytyrosol glucoside, hydroxytyrosol, tyrosol glucoside, and hydroxytyrosol acetate), forms conjugated with secoiridoid derivatives (3,4-DHPEA-EDA and derivatives, oleuropein hexoside isomers, oleuropein isomers, ligustroside, and hydroxytyrosol linked to desoxy elenolic acid), and forms conjugated with hydroxycinnamic acids (verbascoside isomers). Phenolic acids (3-hydroxybenzoic acid and *p*-coumaroyl-6′-secologanoside) and six flavonoids derivatives were also detected.

### 3.4. Effect of Drying

**Figure 5:**
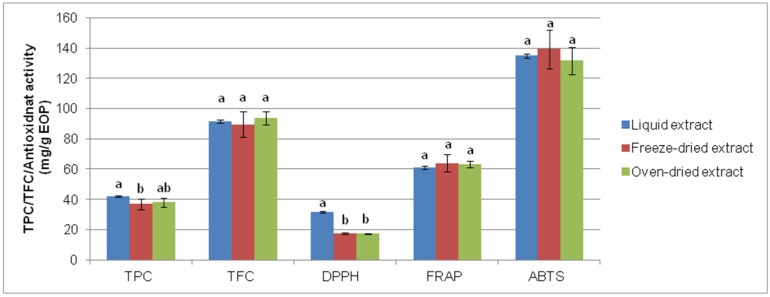
Comparison of freeze-drying and oven-drying of exhausted olive pomace-derived extracts obtained by bath-type ultrasound-assisted extraction: total phenolic content (TPC), total flavonoid content (TFC), and antioxidant activity determined by DPPH, ferric-reducing power (FRAP), and ABTS assays. Data represent the average value and standard deviation (n = 5). For each parameter, bars accompanied by different letters indicate significant differences (*p* < 0.05).

To evaluate the effect of drying, a portion of the extracts obtained after optimal conditions using bath-type UAE was freeze-dried, and another was one oven-dried. The former technique is used to obtain high-quality products in the pharmaceutical and food industry, while the latter is cheaper [49]. Thus, after the redissolution of the dried extracts, the TPC, TFC, and antioxidant activity of these extracts were compared with the values obtained for the liquid extract before drying as a control. Figure 5 shows that the TPC, TFC, and FRAP values were similar after the drying process, and so any of these methods can be chosen to convert the liquid extracts into storable commodities. However, freeze-drying resulted in a dry powdered extract, which was easier to handle.

### 3.5. Chemical Characterization of the Extracted EOP

The solid fraction obtained after UAE, extracted EOP, was chemically characterized for further valorization. As shown in Table 9, the water–acetone extraction step led to the removal of a large portion of the extractives (non-structural components); about 63 and 51.5% of removal using the bath- and probe-type UAE, respectively, when comparing the raw EOP to the solid fraction obtained after extraction. This reduction of the extractive content was similar or slightly lower to that reported using water treatments, with removals of up to 65% (100 °C for 30 min) [2,7]. Additionally, a decrease in the ash content was observed, being aproximately 1.6% in both cases.

Alternatively, the percentage of ethanol extractives increased in the composition; due to 40% acetone’s more polar characteristic, it is probably that the extraction with this solvent was more selective to remove aqueous extractives components than ethanolic ones [50]. The cellulose, hemicellulose, and lignin contents were also increased as a consequence of the partial solubilization of the extractives. This agreed with our previous results when water extraction (85 °C for 90 min) was applied [10], as well as with the results of [2], who applied a water treatment (100 °C for 30 min) for the removal of extractives from EOP. These authors suggested that a first extraction step aimed to remove extractives could be useful in subsequent valorization steps to, for example, valorize sugars, whose contents are enhanced. In any case, the efficiency of the enzymatic hydrolysis obtained to convert cellulose into glucose was low after UAE regardless of the system used, whether it was bath-type UAE (21.6 ± 0.3 g glucose released/100 g glucose in raw EOP) or probe-type UAE (21.1 ± 0.8 g glucose released/100 g glucose in raw EOP). These values were similar to those obtained using water extraction at 85 °C for 90 min (23.7 ± 0.8 g glucose released/100 g glucose in raw EOP). Overall, these results suggest that a pretreatment step of the solid residue would be required after EOP extraction, such as diluted acid and organosolv pretreatments, as the aforementioned studies showed [2,7], in order to enhance this conversion (i.e., polymeric sugars into free sugars) for the further valorization of the sugar fraction.

## 4. Conclusions

The present study revealed that milling of the EOP pellets and UAE favored the extraction of bioactive compounds. A slightly higher TPC was obtained when applying the probe-type UAE as compared to the bath-type UAE for the recovery of these compounds, with TPC values of 45.41 and 42.05 mg GAE/g EOP, respectively. The antioxidant activity and the mannitol content were also higher using the former method; e.g., the manitol content was 69.73 and 59.53 mg/g, respectively. The hydroxytyrosol content was similar after applying these types of extraction, with values of 5.16 and 4.96 mg/g, respectively. In any case, the probe-type UAE was able to shorten the extraction time from 43 to 12 min. This type of extraction shows potential to be applied for obtaining extracts from EOP at the industrial scale in a continuous and faster manner. Moreover, the phenolic profiles of the extracts obtained under optimal conditions using bath- and probe-type UAE were similar after their analysis by CZE-DAD, HPLC-DAD, and HPLC–MS, with hydroxytyrosol being the main component. The extracts showed a similar antioxidant content after freeze-drying and oven-drying, suggesting this type of processes can be applied to obtain storable products for further applications in the food, pharmaceutical, and cosmetic industries—especially using the former technique. Finally, looking for integration in a multiproduct biorefinery process, the extracted EOP solid should be further pretreated if monomeric sugars are desired to be recovered for integral valorization.

## Figures and Tables

**Table 1:** Uncoded and coded values of the factors studied by a Box–Behnken design and a two-level factorial design using bath (B)- and probe (P)-type ultrasound-assisted extraction (UAE), respectively.

Independent Variable	Nomenclature	Units	B-UAE Values			P-UAE Values		
			(−1)	0	(+1)	(−1)	0	(+1)
Acetone concentration	A	%, *v*/*v*	20	50	80	-	-	-
Extraction time	t	min	10	35	60	2	7	12
Solid loading	B	%	2	8.5	15	-	-	-
Amplitude	C	%	-	-	-	30	50	70 ^1^

^1^ Maximum value allowed by the device and ultrasound probe.

**Table 2:** Comparison of the application of milling and ultrasound in the extraction of exhausted olive pomace (EOP): Extraction yield (%), total phenolic content (TPC) (mg gallic acid equivalents/g EOP), total flavonoid content (TFC) (mg rutin equivalents/g EOP), and antioxidant activity (DPPH, FRAP, and ABTS) (mg Trolox equivalents/g EOP). Data represent the average value and standard deviation (n ≥ 3).

	Extraction Yield	TPC	TFC	DPPH	FRAP	ABTS
**Pelletized EOP**						
**Control ^1^**	16.41 ± 1.32 ^c^	11.49 ± 0.11 ^d^	29.35 ± 0.08 ^d^	6.30 ± 0.01 ^d^	14.42 ± 0.21 ^d^	31.01 ± 1.02 ^d^
**UAE**	23.41 ± 1.29 ^b^	18.05 ± 0.80 ^c^	47.13 ± 1.60 ^c^	8.43 ± 0.05 ^c^	25.90 ± 1.35 ^c^	52.04 ± 2.57 ^c^
**Milled EOP**						
**Control ^1^**	43.97 ± 2.18 ^a^	36.69 ± 0.32 ^b^	83.08 ± 1.00 ^b^	31.19 ± 0.10 ^b^	47.59 ± 0.58 ^b^	115.61 ± 0.84 ^b^
**UAE**	46.79 ± 3.63 ^a^	44.59 ± 1.46 ^a^	96.39 ± 2.37 ^a^	34.16 ± 0.33 ^a^	59.27 ± 2.29 ^a^	127.08 ± 4.15 ^a^

Within each column, means with different letters denote statistical significant differences (analysis of variance; *p* < 0.05). ^1^ Extraction at room temperature and without the use of ultrasound.

**Table 3:** Box–Behnken experimental design in terms of actual and coded factors applied to the bath-type ultrasound-assisted extraction of exhausted olive pomace (EOP) and experimental values of the response variables: yield (%), phenolic concentration (PC) (g gallic acid equivalents/L), total phenolic content (TPC) (mg gallic acid equivalents/g EOP), total flavonoid content (TFC) (mg rutin equivalents/g EOP), and antioxidant activity (DPPH, FRAP, and ABTS) (mg Trolox equivalents/g EOP).

Run	A ^1^	t ^1^	B ^1^	T ^2^	Yield	PC	TPC	TFC	DPPH	FRAP	ABTS
1	50 (0)	35 (0)	8.5 (0)	39	50.72	3.94	46.34	96.36	32.46	63.18	136.02
2	20 (−1)	60 (1)	8.5 (0)	41	43.54	3.44	40.44	79.94	30.87	51.53	116.62
3	50 (0)	35 (0)	8.5 (0)	40	45.38	3.69	43.46	94.32	32.09	58.33	130.60
4	50 (0)	10 (−1)	15 (1)	31	43.87	5.78	38.53	86.96	17.31	55.06	119.19
5	50 (0)	10 (−1)	2 (−1)	32	42.87	0.96	48.05	84.95	56.74	49.71	144.78
6	20 (−1)	35 (0)	2 (−1)	39	46.38	0.91	45.32	87.02	55.54	48.88	137.02
7	80 (1)	35 (0)	15 (1)	40	26.84	3.65	24.34	61.65	10.49	31.57	69.69
8	20 (−1)	10 (−1)	8.5 (0)	31	41.65	3.25	38.27	77.71	33.08	47.79	100.62
9	80 (1)	10 (−1)	8.5 (0)	32	28.12	2.05	24.09	54.88	18.74	28.96	69.90
10	20 (−1)	35 (0)	15 (1)	39	43.65	5.68	37.83	80.23	9.99	50.58	110.55
11	50 (0)	35 (0)	8.5 (0)	40	46.47	3.64	42.81	94.27	30.98	58.20	129.85
12	80 (1)	35 (0)	2 (−1)	39	58.62	0.74	36.98	71.80	48.93	37.16	90.98
13	50 (0)	60 (1)	15 (1)	45	46.65	6.52	43.45	93.92	16.48	58.85	119.33
14	50 (0)	35 (0)	8.5 (0)	40	51.65	3.81	44.77	100.04	31.91	59.33	133.37
15	50 (0)	60 (1)	2 (−1)	45	47.48	0.98	49.03	92.16	55.28	52.71	134.88
16	50 (0)	35 (0)	8.5 (0)	40	49.74	3.87	45.58	96.97	33.06	57.34	130.45
17	80 (1)	60 (1)	8.5 (0)	46	36.05	2.64	31.04	74.26	17.88	38.55	83.57

^1^ Factors: A, acetone concentration (%, *v*/*v*); B, solid loading (%, *w*/*v*); t, extraction time (min). ^2^ Temperature (T, °C) reached at the end of the assays.

**Table 4:** Mathematical models and coefficients of the studied responses using coded values for the Box–Behnken design applied to the bath-type ultrasound-assisted extraction of exhausted olive pomace (EOP).

Dependent Variable	Equation No.	Model ^1^	CV (%)	R^2^	Adjusted R^2^	F-Value	*p*-Value	Lack of Fit (*p*-Value)
Extraction yield (%)	(1)	48.79 − 7.66∙A + 1.31∙t − 0.67∙B − 14.50∙A∙B − 3.62∙A − 9.51∙t + 5.94∙B	5.44	0.955	0.901	21.49	<0.0001	0.6938
PC (g GAE/L)	(2)	3.79 − 0.50∙A + 0.19∙t + 2.59∙B − 0.42∙A∙B + 0.18∙t∙B − 0.55∙A − 0.40∙t + 0.17∙B	4.25	0.997	0.994	307.30	<0.0001	0.357
TPC (mg GAE/g EOP)	(3)	44.67 − 6.37∙A + 1.88∙t − 3.71∙B − 10.57∙A	3.72	0.972	0.962	96.64	<0.0001	0.498
TFC (mg RE/g EOP)	(4)	96.27 − 6.99∙A + 4.16∙t + 3.66∙A∙t − 18.72 A − 6.63∙t	2.94	0.971	0.954	59.64	<0.0001	0.423
DPPH (mg TE/g EOP)	(5)	31.60 − 6.67∙A − 0.69∙t − 18.99∙B − 3.25∙A∙B − 5.35∙A − 1.45∙t + 5.41∙B	2.02	0.998	0.997	807.84	<0.0001	0.787
FRAP (mg TE/g EOP)	(6)	57.81 − 7.82∙A + 4.13∙t − 0.67∙B + 1.46∙A∙t − 1.82∙A∙B −3.03∙t∙B − 15.93∙A	2.47	0.993	0.986	140.97	<0.0001	0.193
ABTS (mg TE/g EOP)	(7)	132.06 − 22.74∙A − 1.42∙t − 11.11∙B − 8.40∙A∙t − 37.34∙A − 9.86∙t + 7.34∙B	2.72	0.990	0.981	113.26	<0.0001	0.2584

CV, coefficient of variation; FRAP, ferric-reducing power; GAE, gallic acid equivalents; PC, phenolic concentration; TE, Trolox equivalents; TFC, total flavonoid content; TPC, total phenolic content; RE, rutin equivalents. ^1^ Factors of the model: A, acetone concentration (%, *v*/*v*); B, solid loading (%, *w*/*v*); t, extraction time (min).

**Table 5:** Predicted and experimental values obtained by bath- (B) and probe (P)-ultrasound-assisted extraction (UAE) for exhausted olive pomace (EOP) under the optimal conditions, which simultaneously maximized the seven responses. Data represent the average value and standard deviation (n = 5).

Response Variable	B-UAE			P-UAE		
	Predicted Values	Experimental Values	Error	Predicted Values	Experimental Values	Error
Extraction Yield (%)	49.98	47.32 ± 0.79 ^b^	5.62	59.76	56.79 ± 3.58 ^a^	5.23
Phenolic concentration (g GAE/L)	3.96	3.62 ± 0.05 ^b^	9.39	4.19	3.91 ± 0.27 ^a^	7.16
TPC (mg GAE/g EOP)	46.20	42.05 ± 0.56 ^b^	9.87	48.83	45.41 ± 3.16 ^a^	7.53
TFC (mg RE/g EOP)	96.85	91.59 ± 1.14 ^b^	5.74	111.32	100.81 ± 7.02 ^a^	10.43
DPPH (mg TE/g EOP)	32.54	31.44 ± 0.54 ^b^	3.50	34.69	35.61 ± 0.53 ^a^	2.36
FRAP (mg TE/g EOP)	59.87	61.08 ± 2.23 ^b^	1.98	75.66	68.08 ± 4.32 ^a^	11.13
ABTS (mg TE/g EOP)	137.7	135.0 ± 1.39 ^a^	2.00	147.13	140.67 ± 3.61 ^a^	4.59

FRAP, ferric-reducing power; GAE, gallic acid equivalents; TE, Trolox equivalents; TFC, total flavonoid content; TPC, total phenolic content; RE, rutin equivalents. Within a row, means with different letters denote statistical significant differences among the two types of UAE (*t*-test; *p* < 0.05).

**Table 6:** Factorial experimental design in terms of actual and coded factors applied to the probe-type ultrasound-assisted extraction of exhausted olive pomace (EOP) and experimental values of the response variables: yield (%), phenolic concentration (PC) (g gallic acid equivalents/L), total phenolic content (TPC) (mg gallic acid equivalents/g EOP), total flavonoid content (TFC) (mg rutin equivalents/g EOP), and antioxidant activity (DPPH, FRAP, and ABTS) (mg Trolox equivalents/g EOP).

Run	C ^1^	t ^1^	T ^2^	Yield	PC	TPC	TFC	DPPH	FRAP	ABTS
1	30 (−1)	12 (1)	32	47.09	3.36	39.06	89.37	32.55	59.82	101.89
2	50 (0)	7 (0)	34	44.73	2.98	34.62	87.22	32.55	58.79	115.35
3	70 (1)	12 (1)	50	60.74	4.32	50.27	113.63	35.10	76.73	144.78
4	70 (1)	2 (−1)	28	45.75	3.30	38.38	88.73	32.87	58.58	102.39
5	50 (0)	7 (0)	34	48.71	3.31	38.43	91.48	33.25	61.45	115.85
6	50 (0)	7 (0)	33	46.44	3.20	37.21	85.31	32.62	59.08	112.33
7	30 (−1)	2 (−1)	25	43.39	2.94	34.24	81.31	32.93	53.84	100.51
8	50 (0)	7 (0)	33	47.57	3.25	37.81	87.52	33.14	59.97	119.37
9	50 (0)	7 (0)	33	49.94	3.57	41.46	94.03	31.53	62.34	120.25

^1^ Factors: C, amplitude (%); t, extraction time (min). ^2^ Temperature (T, °C) reached at the end of the assays.

**Table 7:** Mathematical models and coefficients of the studied responses using coded values for the factorial design applied to the probe-type ultrasound-assisted extraction of exhausted olive pomace (EOP).

Dependent Variable	Equation	Model ^1^	CV (%)	R^2^	Adjusted R^2^	F-Value	*p*-Value	Lack of Fit (*p*-Value)
Extraction Yield (%)	(8)	+48.26 + 4.00∙C + 4.67∙t + 2.83∙C∙t	4.46	0.888	0.820	13.18	0.0082	0.262
PC (g GAE/L)	(9)	+3.36 + 0.33∙C + 0.36∙t + 0.15∙C∙t	7.50	0.714	0.6190	7.50	0.0233	0.2207
TPC (mg GAE/g EOP)	(10)	+39.05 + 3.84∙C + 4.18∙t + 1.77∙C∙t	7.50	0.714	0.6190	7.50	0.0233	0.2207
TFC (mg RE/g EOP)	(11)	+90.96 + 7.92∙C + 8.24∙t + 4.21∙C∙t	4.63	0.870	0.792	11.16	0.0112	0.156
DPPH (mg TE/g EOP)	(12)	+32.95 + 0.63∙C + 0.46∙t + 0.65∙C∙t	2.38	0.573	0.316	2.23	0.2022	0.179
FRAP (mg TE/g EOP)	(13)	+61.18 + 5.41∙C + 6.03∙t + 3.04∙C∙t	3.06	0.945	0.912	28.49	0.0014	0.135
ABTS (mg TE/g EOP)	(14)	+182.92 + 17.89∙C + 17.48∙t + 16.38∙C∙t	3.52	0.945	0.912	28.70	0.0014	0.121

CV, coefficient of variation; FRAP, ferric-reducing power; GAE, gallic acid equivalents; PC, phenolic concentration; TE, Trolox equivalents; TFC, total flavonoid content; TPC, total phenolic content; RE, rutin equivalents. ^1^ Factors of the model: C, amplitude (%); t, extraction time (min).

**Table 8:** Phenolic compounds characterized by high-performance chromatography coupled to mass spectrometry in water–acetone extracts obtained by bath- and probe-type ultrasound-assisted extraction under the best obtained conditions.

RT (min)	[M-H]^−^	MS/MS	Compound
Hydroxytyrosol derivatives			
1.1	153	123	Hydroxytyrosol ^1^
1.2	315	153, 135, 123	Hydroxytyrosol hexoside
1.9	299	179, 161, 119, 101	Tyrosol hexoside
6.4	195	153, 151, 59	Hydroxytyrosol acetate
9.3	483	347, 123	Oleacein derivative (+hexose + H_2_)
9.9	623	461, 315	Verbascoside
10.3	701	539, 437, 377, 307, 275	Oleuropein hexoside isomer 1
10.6	335	317, 199, 153	Hydroxy oleacein isomer 1
10.7	623	461	Isoverbascoside
10.9	335	317, 199, 153, 111	Hydroxy oleacein isomer 2
10.6	701	539, 377, 307, 275	Oleuropein hexoside isomer 2
11.4	539	403, 223	Oleouropein isomer 1
11.6	539	403, 377, 307, 275, 223	Oleuropein ^1^
11.8	701	377, 307, 275	Oleuropein hexoside isomer 3
12.4	539	377, 307, 275, 223	Oleouropein isomer 2
12.7	539	403, 377, 307, 275, 223	Oleouropein isomer 3
13.1	319	183, 181, 153, 111	3,4-DHPEA-EDA ^2^ or oleacein
13.5	523	361, 291, 259, 223	Ligustroside
18.7	361	329, 291, 225, 193, 181	Hydroxytyrosol linked to desoxy elenolic acid
Non-hydroxytyrosol derivatives			
6.0	137	Not fragmented	Hydroxybenzoic acid
9.7	463	347, 301	Quercetin hexoside
10.0	447	285	Luteolin 7-*O*-glucoside ^1^
10.2	593	285	Luteolin *O*-deoxyhexosyl-hexoside
10.5	593	447, 285	Luteolin *O*-deoxyhexoside *O*-hexoside
11.7	551	507, 389, 341, 281, 251, 179	Caffeoyl-6′-secologanoside
13.2	535	491, 389, 345, 265, 163	*p*-Coumaroyl-6′-secologanoside
14.2	285	175, 151	Luteolin

^1^ Compared with standards. ^2^ 3,4-DHPEA-EDA (decarboxymethyl oleuropein aglycone).

**Table 9:** Chemical composition of the exhausted olive pomace (EOP) before and after bath (B)- and probe (P)-ultrasound assisted extraction (UAE). Data (%, dry weight basis) represent the average value and standard deviation (n = 3).

Component	Raw EOP ^2^	B-UAE	P-UAE
Extractives	42 ± 2	15.5 ± 0.4	20 ± 1
Aqueous extractives	38 ± 2	4.9 ± 0.2	8.0 ± 1
Ethanol extractives	3.8 ± 0.2	10.8 ± 0.4	12 ± 1
Cellulose	9.7 ± 0.8	11.5 ± 0.7	15.1 ± 0.8
Hemicellulose	10.9 ± 0.5	15.8 ± 0.8	20.1 ± 0.9
Xylan ^1^	9.8 ± 0.5	11.3 ± 0.8	15.8 ± 0.9
Galactan ^1^	0.3 ± 0.3	2.84 ± 0.09	2.6 ± 0.6
Arabinan ^1^	1.82 ± 0.03	2.27 ± 0.02	2.76 ± 0.01
Mannan ^1^	0.42 ± 0.02	1.3 ± 0.2	1.41 ± 0.03
Acetyl groups	1.5 ± 0.2	1.4 ± 0.2	1.84 ± 0.05
Lignin	21.8 ± 0.9	30.1 ± 0.3	29.79 ± 0.07
Acid insoluble lignin	20.3 ± 0.7	29.6 ± 0.3	28.5 ± 0.7
Acid soluble lignin	1.5 ± 0.5	0.55 ± 0.06	1.27 ± 0.07
Ash	6.4 ± 0.2	1.67 ± 0.06	1.6 ± 0.2

^1^ Hemicellulosic sugars. ^2^ Data from Gómez-Cruz et al. [10].

## Data Availability

Not applicable.

## Supplementary Materials

The following are available online at https://www.mdpi.com/article/10.3390/biology10060514/s1. Table S1. Final temperature reached in the experimental assays of the Box–Behnken design and factorial design for the bath- and probe-type ultrasound-assisted extraction (UAE), respectively. Table S2. F-ratios and *p*-values obtained for the operational parameters when the increment of temperature was evaluated as response variable in the Box–Behnken design (BBD) and in the factorial design (FD) for the bath- and probe-type ultrasound-assisted extraction, respectively. Figure S1. Response surface plot for the increment of temperature as function (a) of the time and acetone in the Box–Behnken design and (b) of time and amplitude in the factorial design. Figure S2. HPLC chromatogram at 280 nm of the exhaustive olive pomace extract obtained with water–acetone at the best conditions by: (a) bath- and (b) probe (P)-type ultrasound-assisted extraction. * Acetone signal. Figure S3. Base peak chromatogram of the exhaustive olive pomace extract obtained with water–acetone at optimal conditions by: (a) bath- and (b) probe-type ultrasound-assisted extraction. The characterized compounds are also shown.

## References

[1] Contreras M.d.M., Romero I., Moya M., Castro E. (2020). Olive-derived biomass as a renewable source of value-added products. Process Biochem..

[2] Manzanares P., Ballesteros I., Negro M.J., González A., Oliva J.M., Ballesteros M. (2020). Processing of extracted olive oil pomace residue by hydrothermal or dilute acid pretreatment and enzymatic hydrolysis in a biorefinery context. Renew. Energy.

[3] López-Linares J.C., Gómez-Cruz I., Ruiz E., Romero I., Castro E. (2020). Production of ethanol from hemicellulosic sugars of exhausted olive pomace by *Escherichia coli*. Processes.

[4] De La Casa J.A., Castro E. (2014). Recycling of washed olive pomace ash for fired clay brick manufacturing. Constr. Build. Mater..

[5] Ruiz E., Romero-García J.M., Romero I., Manzanares P., Negro M.J., Castro E. (2017). Olive-derived biomass as a source of energy and chemicals. Biofuels Bioprod. Biorefin..

[6] López-Linares J.C., Ruiz E., Romero I., Castro E., Manzanares P. (2020). Xylitol production from exhausted olive pomace by *Candida boidinii*. Appl. Sci..

[7] Gómez-Cruz I., Contreras M.d.M., Romero I., Castro E. (2021). A biorefinery approach to obtain antioxidants, lignin and sugars from exhausted olive pomace. J. Ind. Eng. Chem..

[8] Leite P., Salgado J.M., Venâncio A., Domínguez J.M., Belo I. (2016). Ultrasounds pretreatment of olive pomace to improve xylanase and cellulase production by solid-state fermentation. Bioresour. Technol..

[9] Dermeche S., Nadour M., Larroche C., Moulti-Mati F., Michaud P. (2013). Olive mill wastes: Biochemical characterizations and valorization strategies. Process Biochem..

[10] Gómez-Cruz I., Cara C., Romero I., Castro E., Gullón B. (2020). Valorisation of exhausted olive pomace by an ecofriendly solvent extraction process of natural antioxidants. Antioxidants.

[11] Contreras M.d.M., Gómez-Cruz I., Romero I., Castro E. (2021). Olive pomace-derived biomasses fractionation through a chemical characteristics. Foods.

[12] Mosca A., Crudele A., Smeriglio A., Braghini M.R., Panera N., Comparcola D., Alterio A., Sartorelli M.R., Tozzi G., Raponi M. (2020). Antioxidant activity of Hydroxytyrosol and Vitamin E reduces systemic inflammation in children with paediatric NAFLD. Dig. Liver Dis..

[13] Lopez-Huertas E., Fonolla J. (2017). Hydroxytyrosol supplementation increases vitamin C levels in vivo. A human volunteer trial. Redox Biol..

[14] Bellumori M., Cecchi L., Innocenti M., Clodoveo M.L., Corbo F., Mulinacci N. (2019). The EFSA health claim on olive oil polyphenols: Acid hydrolysis validation and total hydroxytyrosol and tyrosol determination in Italian virgin olive oils. Molecules.

[15] Suárez M., Romero M.P., Ramo T., Motilva M.J. (2011). Stability of a phenol-enriched olive oil during storage. Eur. J. Lipid Sci. Technol..

[16] Romeo R., De Bruno A., Imeneo V., Piscopo A., Poiana M. (2020). Impact of stability of enriched oil with phenolic extract from olive mill wastewaters. Foods.

[17] Aliakbarian B., Casazza A.A., Perego P. (2011). Valorization of olive oil solid waste using high pressure-high temperature reactor. Food Chem..

[18] Şahin S., Aybastier Ö., Işik E. (2013). Optimisation of ultrasonic-assisted extraction of antioxidant compounds from *Artemisia absinthium* using response surface methodology. Food Chem..

[19] Lama-Muñoz A., Contreras M.d.M., Espínola F., Moya M., Romero I., Castro E. (2019). Optimization of oleuropein and luteolin-7-O-glucoside extraction from olive leaves by ultrasound-assisted technology. Energies.

[20] Azmir J., Zaidul I.S.M., Rahman M.M., Sharif K.M., Mohamed A., Sahena F., Jahurul M.H.A., Ghafoor K., Norulaini N.A.N., Omar A.K.M. (2013). Techniques for extraction of bioactive compounds from plant materials: A review. J. Food Eng..

[21] Muñiz-Márquez D.B., Martínez-Ávila G.C., Wong-Paz J.E., Belmares-Cerda R., Rodríguez-Herrera R., Aguilar C.N. (2013). Ultrasound-assisted extraction of phenolic compounds from *Laurus nobilis* L. and their antioxidant activity. Ultrason. Sonochem..

[22] Zeković Z., Pintać D., Majkić T., Vidović S., Mimica-Dukić N., Teslić N., Versari A., Pavlić B. (2017). Utilization of sage by-products as raw material for antioxidants recovery—Ultrasound versus microwave-assisted extraction. Ind. Crops Prod..

[23] Medina-Torres N., Ayora-Talavera T., Espinosa-Andrews H., Sánchez-Contreras A., Pacheco N. (2017). Ultrasound assisted extraction for the recovery of phenolic compounds from vegetable sources. Agronomy.

[24] Cikoš A.M., Jokić S., Šubarić D., Jerković I. (2018). Overview on the application of modern methods for the extraction of bioactive compounds from marine macroalgae. Mar. Drugs.

[25] Vilkhu K., Mawson R., Simons L., Bates D. (2008). Applications and opportunities for ultrasound assisted extraction in the food industry—A review. Innov. Food Sci. Emerg. Technol..

[26] Yao L., Xiong L., Yoo C.G., Dong C., Meng X., Dai J., Ragauskas A.J., Yang C., Yu J., Yang H. (2020). Correlations of the physicochemical properties of organosolv lignins from *Broussonetia papyrifera* with their antioxidant activities. Sustain. Energy Fuels.

[27] Sluiter A., Hames B., Ruiz R., Scarlata C., Sluiter J., Templeton D., Crocker D. (2012). Determination of Structural Carbohydrates and Lignin in Biomass Determination of Structural Carbohydrates and Lignin in Biomass.

[28] Singleton V.L., Rossi S.A. (1965). Colorimetric of total phenolics with phosphomolibic Phosphotungstic acid reagents. Am. J. Enol. Vitic..

[29] Blasa M., Candiracci M., Accorsi A., Piacentini M.P., Albertini M.C., Piatti E. (2006). Raw Millefiori honey is packed full of antioxidants. Food Chem..

[30] Martínez-Patiño J.C., Gullón B., Romero I., Ruiz E., Brnčić M., Žlabur J.Š., Castro E. (2019). Optimization of ultrasound-assisted extraction of biomass from olive trees using response surface methodology. Ultrason. Sonochem..

[31] Medfai W., Contreras M.d.M., Lama-Muñoz A., Mhamdi R., Oueslati I., Castro E. (2020). How cultivar and extraction conditions affect antioxidants type and extractability for olive leaves valorization. ACS Sustain. Chem. Eng..

[32] Gómez-Cruz I., Romero I., Contreras M., Padilla-rascón C., Carvalheiro F., Duarte L.C., Roseiro L.B. (2021). Exhausted olive pomace phenolic-rich extracts obtention: A first step for a biorefinery scheme proposal. Proceedings.

[33] Royal Decree 1101/2011 (2011). Real Decreto 1101/2011, de 22 de julio, por el que se aprueba la lista positiva de los disolventes de extracción que se pueden utilizar en la fabricación de productos alimenticios y de sus ingredientes. BOE.

[34] Kumar K., Srivastav S., Sharanagat V.S. (2021). Ultrasound assisted extraction (UAE) of bioactive compounds from fruit and vegetable processing by-products: A review. Ultrason. Sonochem..

[35] Wani S.A., Bishnoi S., Kumar P. (2016). Ultrasound and microwave assisted extraction of diosgenin from fenugreek seed and fenugreek-supplemented cookies. J. Food Meas. Charact..

[36] Kaleem M., Ahmad A., Amir R.M., Raja G.K. (2019). Ultrasound-assisted phytochemical extraction condition optimization using response surface methodology from perlette grapes (*Vitis vinifera*). Processes.

[37] Jun H.I., Song G.S., Yang E.I., Youn Y., Kim Y.S. (2012). Antioxidant Activities and phenolic compounds of pigmented rice bran extracts. J. Food Sci..

[38] Mokrani A., Madani K. (2016). Effect of solvent, time and temperature on the extraction of phenolic compounds and antioxidant capacity of peach (*Prunus persica* L.) fruit. Sep. Purif. Technol..

[39] Wijekoon M.M.J.O., Bhat R., Karim A.A. (2011). Effect of extraction solvents on the phenolic compounds and antioxidant activities of bunga kantan (*Etlingera elatior* Jack.) inflorescence. J. Food Compos. Anal..

[40] Medina E., Romero C., García P., Brenes M. (2019). Characterization of bioactive compounds in commercial olive leaf extracts, and olive leaves and their infusions. Food Funct..

[41] Cádiz-Gurrea M.D., Pinto D., Delerue-Matos C., Rodrigues F. (2021). Olive fruit and leaf wastes as bioactive ingredients for cosmetics—A preliminary study. Antioxidants.

[42] Carrera C., Ruiz-Rodríguez A., Palma M., Barroso C.G. (2012). Ultrasound assisted extraction of phenolic compounds from grapes. Anal. Chim. Acta.

[43] Rohilla S., Mahanta C.L. (2021). Optimization of extraction conditions for ultrasound-assisted extraction of phenolic compounds from tamarillo fruit (*Solanum betaceum*) using response surface methodology. J. Food Meas. Charact..

[44] Zardo I., de Espíndola Sobczyk A., Marczak L.D.F., Sarkis J. (2019). Optimization of Ultrasound Assisted Extraction of phenolic compounds from sunflower seed cake using response surface methodology. Waste Biomass Valorization.

[45] Ribeiro T.B., Oliveira A.L., Costa C., Nunes J., Vicente A.A., Pintado M. (2020). Total and sustainable valorisation of olive pomace using a fractionation approach. Appl. Sci..

[46] Rigane G., Bouaziz M., Baccar N., Abidi S., Sayadi S., Salem R. (2012). Recovery of hydroxytyrosol rich extract from two-phase chemlali olive pomace by chemical treatment. J. Food Sci..

[47] Lama-Muñoz A., Contreras M.d.M., Espínola F., Moya M., Romero I., Castro E. (2020). Content of phenolic compounds and mannitol in olive leaves extracts from six Spanish cultivars: Extraction with the Soxhlet method and pressurized liquids. Food Chem..

[48] Ammar S., Contreras M.d.M., Gargouri B., Segura-Carretero A., Bouaziz M. (2017). RP-HPLC-DAD-ESI-QTOF-MS based metabolic profiling of the potential *Olea europaea* by-product “wood” and its comparison with leaf counterpart. Phytochem. Anal..

[49] Kawasaki H., Shimanouchi T., Kimura Y. (2019). Recent development of optimization of lyophilization Process. J. Chem..

[50] Musa K.H., Abdullah A., Jusoh K., Subramaniam V. (2011). Antioxidant activity of pink-flesh guava (*Psidium guajava* L.): Effect of Extraction techniques and solvents. Food Anal. Methods.

