# Supplementary material for: Recovery of Bioactive Compounds from Industrial Exhausted Olive Pomace through Ultrasound-Assisted Extraction
Source: Biology (Basel). 2021 Jun 10;10(6):514. doi: 10.3390/biology10060514 (PMC8229622; doi:10.3390/biology10060514)
Supplement: Supplementary file 1 [file biology-10-00514-s001.zip › biology-1222264-supplementary.pdf]

Table S1. Final temperature reached in the experimental assays of the Box-Benhken design and factorial design for the bath- and probe-type ultrasound-assisted extraction (UAE), respectively.

RUN	Increment of Temperature (°C)	RUN	Increment of Temperature (°C)
Box-Benhken design		14	14
1	11	15	19
2	19	16	15
3	12	17	19
4	4	Factorial design	
5	5	1	8
6	12	2	10
7	13	3	26
8	4	4	4
9	5	5	10
10	11	6	9
11	13	7	1
12	11	8	9
13	18	9	8

Table S2. F-ratios and p-values obtained for the operational parameters when the increment of temperature was evaluated as response variable in the Box-Behnken design (BBD) and in the factorial design (FD) for the bath- and probe-type ultrasound-assisted extraction, respectively.

**Increment of Temperature (°C) (BBD)**		
Source	F-Ratio	P-Value
A:Acetone	1.04	0.3411
t:Time	376.54	<0.0001
B:Solids	0.46	0.5178
AA	0.88	0.3792
At	0.23	0.6449
AB	0.93	0.3677
tt	3.71	0.0954
tB	0	1
BB	2.05	0.1951
**Increment of temperature (°C) (FD)**		
Source	F-Ratio	P-Value
C:Amplitude	158.76	<0.0001
t:time	302.76	<0.0001
Ct	81	0.0003

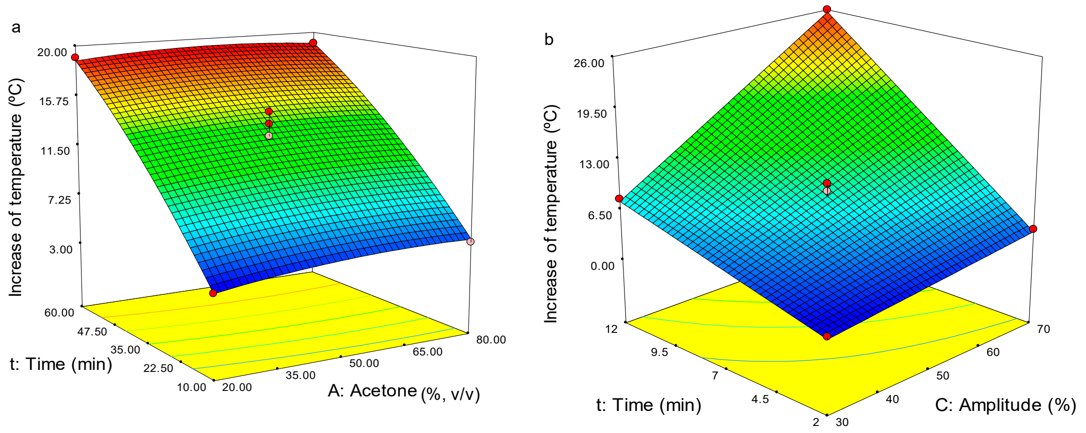

Figure S1. Response surface plot for the increment of temperature as function a) of the time and acetone in the Box-Behnken design and b) of time and amplitude in the factorial design.

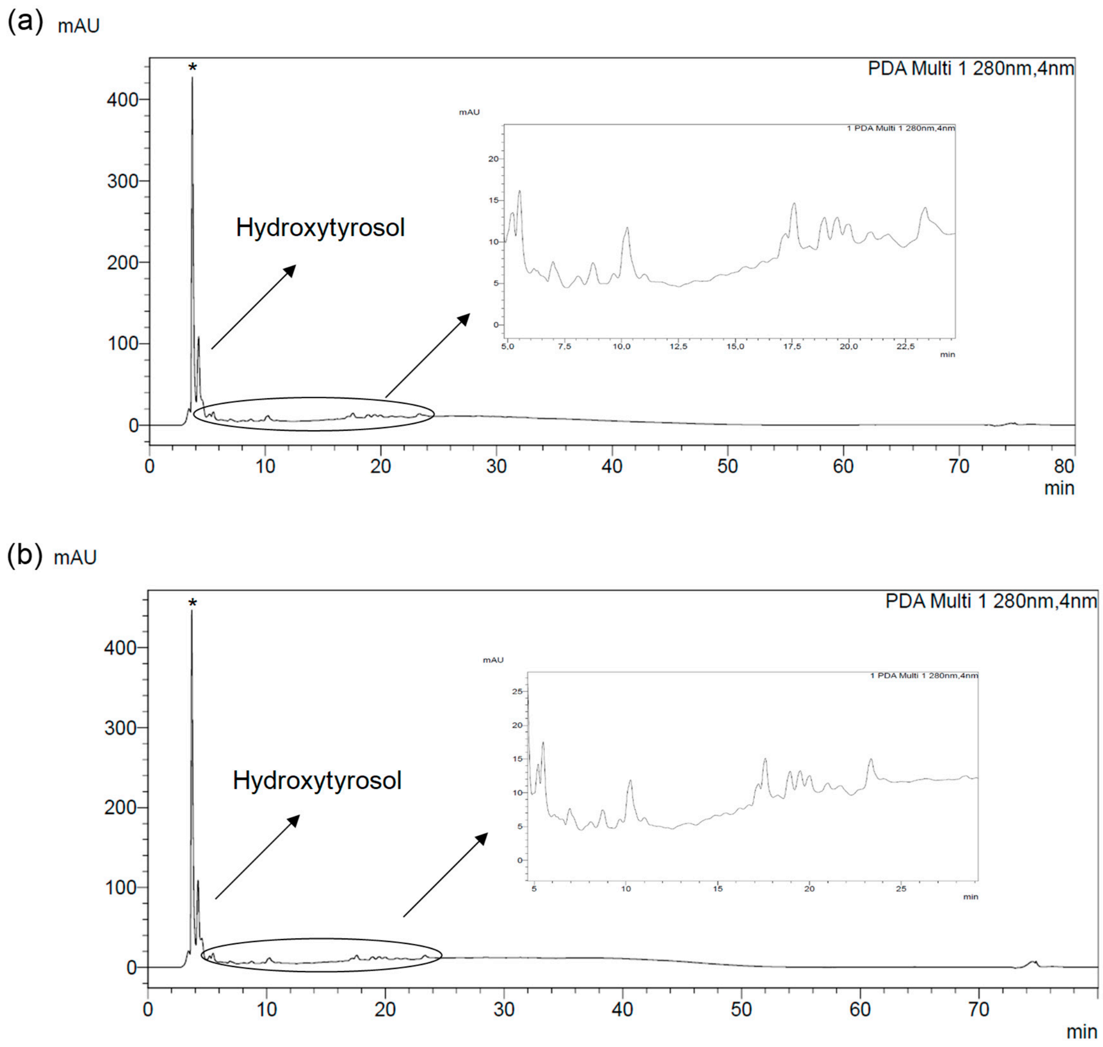

Figure S2. HPLC chromatogram at 280 nm of the exhaustive olive pomace extract obtained with water-acetone at optimal conditions by: (**a**) bath- and (**b**) probe-type ultrasound-assisted extraction. * Acetone signal.

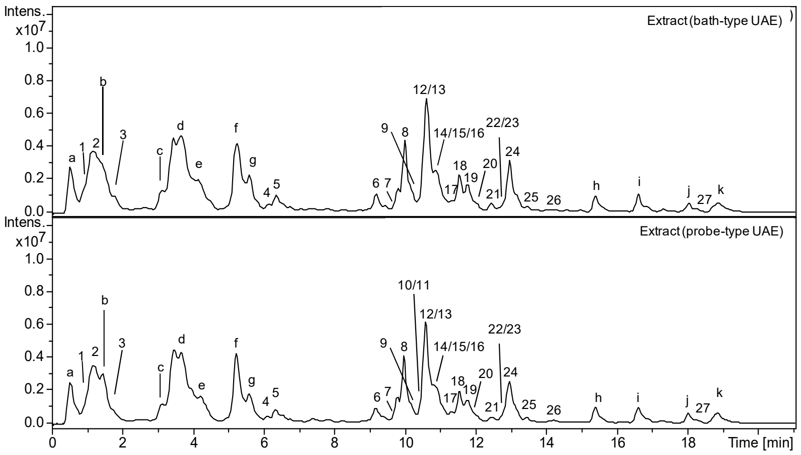

No	Hydroxytyrosol derivatives	No	Hydroxytyrosol derivatives	No	Non-hydroxytyrol derivatives	Letter	Non-phenolic compounds
1	Hydroxytyrosol	15	Oleuropein hexoside isomer 2	4	3-Hydroxybenzoic acid	a	Mannitol, citric acid
2	Hydroxytyrosol hexoside	16	Hydroxy oleacein isomer 2	7	Quercetin hexoside	b	Loganin derivative (+H_2; +O)
3	Tyrosol hexoside	17	Oleouropein isomer 1	9	Luteolin 7-*O*-glucoside	c	Oleoside/secologanoside derivative (-glucosyl; -CO_2) or decarboxymethylelenolic acid
5	Hydroxytyrosol acetate	19	Oleuropein	11	Luteolin *O*-deoxyhexosyl-hexoside	d	Oleoside/Secologanoside
6	Oleacein derivative (+ hexose + H_2)	20	Oleuropein hexoside isomer 3	12	Luteolin O-deoxyhexoside *O*-hexoside	e	Loganin derivative (+H_2; +O; +CO)
8	Verbascoside	21	Oleouropein isomer 2	18	Caffeoyl-6'-secologanoside	f	Elenolic acid hexoside derivative (+H_2; -CO)
10	Oleuropein hexoside isomer 1	22	Oleouropein isomer 3	24	*p*-coumaroyl-6'-secologanoside	g	Unknown
13	Hydroxy oleacein isomer 1	23	3,4-DHPEA-EDA[2] or oleacein	26	Luteolin	h	6'-*O*-[(2E)-2,6-Dimethyl-8-hydroxy-2-octenoyloxy]-secologanoside
14	Isoverbascoside	25	Ligustroside			i	Trihydroxyoctadecenoic acid
		27	Hydroxytyrosol linked to desoxy elenolic acid			j	Trihydroxyoctadecanoic acid
						k	Dihydroxyhexadecanoic acid

Figure S3. Base peak chromatogram of the exhaustive olive pomace extract obtained with water-acetone at optimal conditions by: (**a**) bath- and (**b**) probe-type ultrasound-assisted extraction. The characterized compounds are also shown.
